# Impact of Nephrology Consultation on Moderate and Severe Hyponatremia Outcomes and Length of Hospital Stay

**DOI:** 10.7759/cureus.99495

**Published:** 2025-12-17

**Authors:** Zainulabdeen S Al-saedi, Mohammed A Miqdad, Lina Alatta, Hasan Hulwi, Oscar Rodriguez, Sarah Alshahban, Bruce Spinowitz

**Affiliations:** 1 Medicine/Nephrology, NewYork-Presbyterian Queens Hospital, Flushing, USA; 2 Research, Michigan State University, Michigan, USA; 3 Nephrology, NewYork-Presbyterian Queens Hospital, Flushing, USA; 4 Internal Medicine/Nephrology, NewYork-Presbyterian Queens Hospital, Flushing, USA; 5 Internal Medicine, Baghdad Medical City, Baghdad, IRQ

**Keywords:** electrolyte disorders, hospital readmission, hyponatremia, inpatient outcomes, length of stay, mortality, nephrology consultation, osmotic demyelination syndrome, retrospective cohort, sodium correction

## Abstract

Background

Hyponatremia is a frequent electrolyte disorder among hospitalized patients, associated with increased morbidity, mortality, and healthcare costs. Timely nephrology consultation may influence patient outcomes, but its impact remains unclear.

Objective

To evaluate the association of early versus late or no nephrology consultation on clinical outcomes, including mortality, length of stay, sodium correction rates, and readmission among hospitalized patients with moderate to severe hyponatremia.

Methods

We conducted a retrospective cohort study of 423 adult patients admitted with serum sodium <130 mmol/L at New York-Presbyterian Queens from July 2023 to June 2024. Patients were categorized into early consultation (≤48 hours), late consultation (>48 hours), or no nephrology consultation groups. Primary outcomes included inpatient, 30-day, and 90-day mortality, and length of hospital stay. Secondary outcomes comprised readmission rates, discharge disposition, recurrence of hyponatremia, and neurological complications, including osmotic demyelination syndrome. Multivariable regression adjusted for confounders such as age, comorbidities, and admission unit.

Results

Early nephrology consultation was provided to 41% of patients, late consultation to 20%, and 39% received no consultation. Mortality rates did not differ significantly among groups at any time point. Nephrology consultation was associated with a longer hospital stay (median 7-8 days vs. 5 days; p<0.001) but did not significantly affect sodium correction rates or readmission. No cases of osmotic demyelination syndrome were observed. Causes of death were predominantly cancer, infection, and cardiorespiratory events. Multivariable analysis confirmed no significant mortality difference attributable to consultation timing but a significant increase in length of stay with nephrology involvement.

Conclusion

In patients with moderate to severe hyponatremia, nephrology consultation was associated with longer hospitalization but did not significantly alter mortality or readmission rates. Further prospective studies are needed to clarify the role of nephrology input in optimizing outcomes and resource utilization in hyponatremic patients.

## Introduction

Hyponatremia is defined as a serum sodium level of less than 135 milliequivalents per liter (mEq/L) and is the most common electrolyte abnormality, with a prevalence of 20% to 35% among hospitalized patients. It represents a relative water-to-sodium imbalance, where total body water exceeds total body solutes [1]. Hyponatremia is associated with increased all-cause mortality across various clinical settings, and patients with severe hyponatremia (serum sodium <120 mEq/L) have a hospital mortality rate that is twice as high as that of patients with normal sodium levels (eunatremia) [2].

Severe hyponatremia can lead to hyponatremic encephalopathy, which requires urgent treatment with hypertonic saline to prevent fatal or permanent neurological consequences. United States (US) and European guidelines set limits on sodium correction rates to prevent osmotic demyelination syndrome (ODS), but these recommendations are based on low-quality evidence and expert consensus [3].

Profound hyponatremia (plasma sodium <125 mmol/L) is relatively rare, with a prevalence of 0.15-2.5%. It is associated with increased morbidity and mortality in conditions such as heart failure, myocardial infarction, liver cirrhosis, pneumonia, chronic obstructive pulmonary disease (COPD), and stroke. Additionally, it increases the risk of falls, fractures, and osteoporosis. Profound hyponatremia often requires Intensive Care Unit (ICU) admission and is an independent risk factor for poor prognosis, rehospitalization, and mortality. Acute symptomatic hyponatremia requires urgent treatment, whereas overcorrection in chronic cases can lead to osmotic demyelination. Proper diagnosis is crucial for successful management, but identifying the underlying cause remains challenging, with studies highlighting deficits in hospital-based management [4].

Correcting hyponatremia has been linked to reduced mortality, with benefits that can last up to a year after discharge. However, rapid correction in patients whose bodies have adapted to low sodium levels is associated with osmotic demyelination syndrome (ODS), a rare but potentially fatal condition. The relationship between sodium correction rates and ODS risk remains uncertain. Some studies suggest that ODS is more likely when sodium levels increase by more than 12 mEq/L within 24 hours, but others have found no clear association [5].

The relationship between hyponatremia correction rates and mortality is complex, making it difficult to determine whether observed associations are due to physician interventions or the severity of patients' underlying conditions. Some studies suggest that rapid sodium correction may be linked to higher mortality, but this could reflect the severity of comorbidities rather than the correction itself. For instance, in a retrospective study, only 12% of patients received hypertonic saline, indicating that spontaneous water diuresis, rather than active treatment, often caused sodium correction [6].

In 1938, Helwig et al. first used rapid infusion of hypertonic saline to treat a moribund patient, with complete recovery [7]*.* This approach has since been employed by others in patients with severe hyponatremia, resulting in complete recovery without evidence of central pontine myelinolysis (CPM) [2,7,8].

Patients with severe conditions such as decompensated cirrhosis, advanced heart failure, metastatic cancer, or severely reduced kidney function tend to have slower sodium correction due to sustained vasopressin secretion and impaired urine dilution. In contrast, patients with reversible causes of hyponatremia - such as hypovolemia, transient Syndrome of Inappropriate Antidiuretic Hormone (SIADH), adrenal insufficiency, or thiazide-induced hyponatremia - are more likely to experience rapid correction due to spontaneous water diuresis, which is generally associated with better outcomes [6].

ODS can occur even when appropriate hyponatremia correction rates are followed. Risk factors for ODS include a sodium level ≤ 105 mEq/L, hypokalemia, malnutrition, alcohol use disorder, and liver disease [9,10]. Osmotic demyelination is a neuropsychiatric condition first described by Adams et al., characterized by demyelination in the basal pons and other brain areas such as the basal ganglia, thalamus, and cerebellum [12]*.* Pathologically, it involves the loss of oligodendrocytes and myelin, while neurons and axons are preserved. While ODS can cause severe symptoms like locked-in syndrome, many cases are mild or asymptomatic. Rapid correction of chronic hyponatremia is the primary risk factor, as the brain adapts to low sodium levels over time. If hyponatremia is corrected too quickly, the brain's protective mechanisms fail, leading to osmotic stress, cell dehydration, and disruption of the blood-brain barrier, which can further damage oligodendrocytes [11,12].

Changes in medical practices have made osmotic demyelination syndrome less common since the 1980s. Current guidelines recommend avoiding a sodium correction of ≥10 mmol/L within 24 hours or ≥18 mmol/L within 48 hours. Although rapid correction does not always cause ODS, it increases the risk. The longer the duration of hyponatremia and the lower the sodium level, the higher the risk of injury from rapid correction. If the sodium level is ≤105 mmol/L or if other risk factors for ODS are present, extra caution is needed [13].

Hyponatremia is associated with significant clinical outcomes, including increased mortality, hospital readmission rates, and higher healthcare costs. Severe hyponatremia, particularly with sodium levels below 120 mEq/L, is a strong predictor of mortality. Studies have shown that patients with low sodium levels face an increased risk of both short-term and long-term mortality, with death often due to complications such as neurological impairment or comorbid conditions like heart failure and liver cirrhosis [14].

Additionally, hyponatremia is associated with higher rates of hospital readmissions, particularly in patients with chronic hyponatremia or those who experience insufficient correction during the initial hospitalization. These readmissions are primarily driven by recurrent hyponatremia episodes and complications related to its management [15].

The economic burden of hyponatremia is considerable, stemming from extended hospital stays, diagnostic tests, and treatment for associated complications such as osmotic demyelination syndrome [16,17].

Timely consultation with a nephrologist is crucial in managing patients with severe hyponatremia, rapidly decreasing sodium levels, or persistent hyponatremia [1]. A six-month study at a tertiary hospital in the Philippines evaluated the impact of referral timing on outcomes in 800 hyponatremic patients. The study revealed that earlier nephrology referrals were associated with shorter hospital stays and lower mortality rates. Overall mortality was 31.87%, with those referred within the first day having a median hospital stay of 8.41 days and a mortality rate of 26.04%. In contrast, patients referred after a week had a prolonged hospital stay of 24.57 days and a mortality rate of 51.35% (HR = 2.91; 95% CI, 1.94-4.36). Patients referred between 3 to 7 days had a higher mortality risk compared to those referred within the first day [18].

## Materials and methods

Study setting

We conducted a single-center retrospective cohort study at New York-Presbyterian Queens (NYPQ) Hospital, a tertiary-care facility in Flushing, Queens, New York City. NYPQ serves a diverse patient population across Queens County, providing comprehensive medical care, including specialized nephrology services. This study focused on adult patients admitted with hyponatremia between July 1, 2023, and June 30, 2024. The Institutional Review Board (IRB) of NYPQ (IRB file number 16250924) approved the study and authorized the use of patient data for research purposes. Given the retrospective nature of the study and the absence of direct patient interaction, the IRB waived the requirement for written informed consent.

Data source

Patient data were extracted from the Electronic Medical Record (EMR) system, EPIC, using a structured chart review. A query within the i2b2 database was used to identify eligible cases based on ICD-10 diagnostic codes and laboratory values. The study included all patients admitted under the general internal medicine floor (GMF), including those initially admitted to the ICU before transferring to GMF. Demographic, clinical, laboratory, and outcome data were systematically collected.

Study population

**Figure 1:**
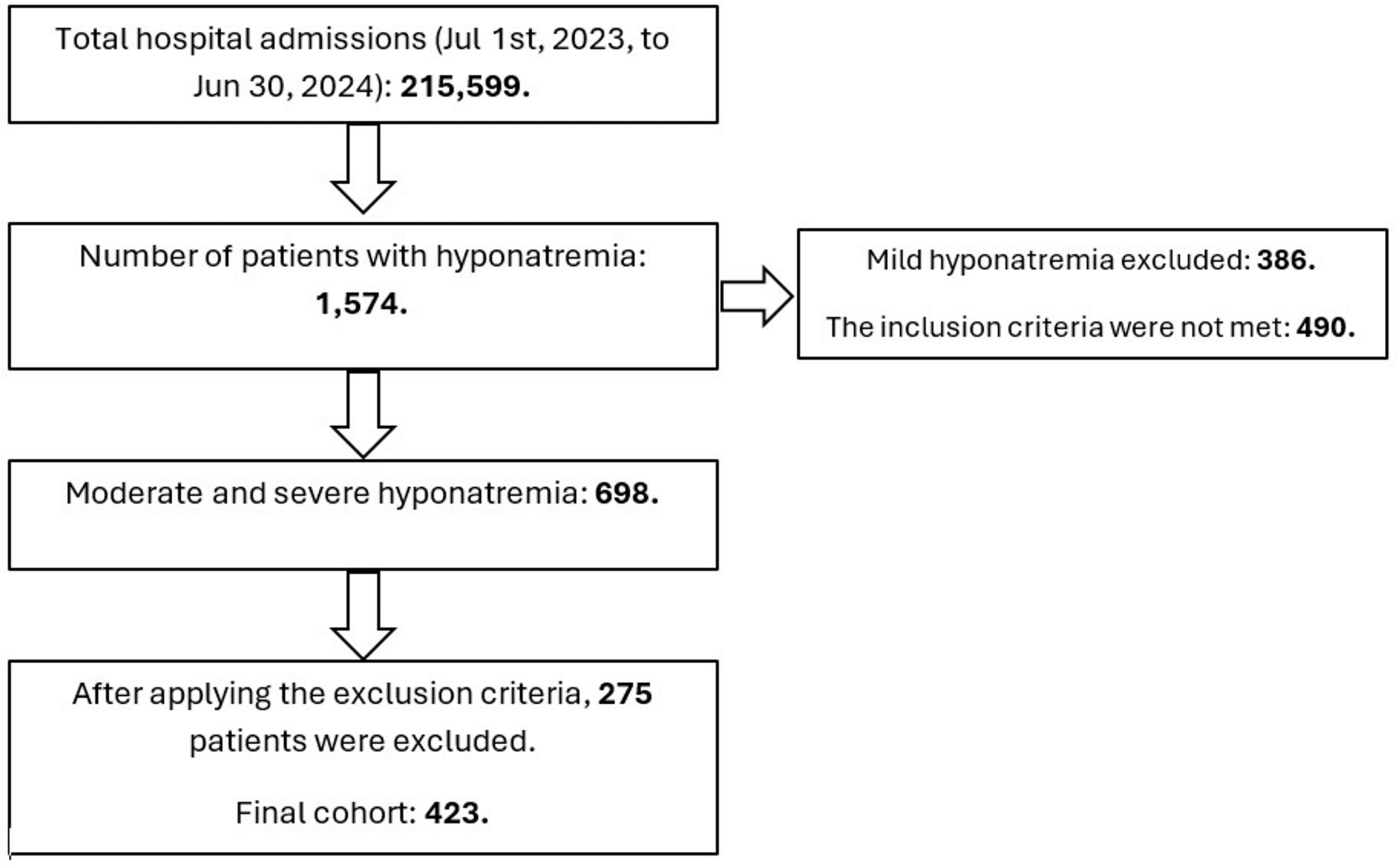
Flow Diagram of Patients' Inclusion

We identified all adult (age ≥18 years) admissions with moderate to severe hyponatremia, defined as an initial serum sodium level (Na) <130 mmol/L upon emergency department (ED) presentation. To ensure a uniform cohort, we excluded patients who developed hyponatremia during hospitalization, as this represents a distinct clinical entity. Patients discharged directly from the ED without inpatient admission were also excluded (Figure 1).

If a patient had multiple admissions meeting the inclusion criteria within the study period, each eligible admission was analyzed separately. Patients with an initial blood glucose level ≥25 mmol/L were excluded due to the potential for hyperglycemia-induced pseudohyponatremia.

Inclusion and exclusion criteria

Patients were eligible for inclusion if they were ≥18 years old, admitted to NYPQ general medicine or ICU service between July 1, 2023, and June 30, 2024, had a diagnosis of hyponatremia (ICD-10: E87.1) documented during the ED or inpatient encounter, and had an initial serum sodium <130 mmol/L at the time of ED presentation. Patients were excluded if they had chronic kidney disease (CKD) stage 3-5 or end-stage renal disease (ICD-10: N18.3, N18.4, N18.5, N18.6), had an initial serum sodium ≥130 mmol/L (sodium corrected to glucose using the formula: $\mathrm{Na}_{\mathrm{corrected}} = \mathrm{Na}_{\mathrm{measured}} + 0.016 \times (\mathrm{Glucose} - 100)$ [19], were discharged directly from the ED without inpatient admission, or were referred to hospice or comfort care measures.

Definitions

Hyponatremia was defined as a serum sodium concentration <130 mmol/L at the time of ED presentation [20]. Consultation referred to a formal nephrology consultation requested by the primary medical team to assess and manage hyponatremia in hospitalized patients. Early Consultation was defined as a nephrology consultation initiated within 48 hours of hospital admission, while Late Consultation referred to consultations initiated more than 48 hours after hospital admission. Sodium correction rate over a 24-hour period was categorized as slow if less than 6 mEq/L, average if between 6 and 10 mEq/L, and rapid if greater than 10 mEq/L. Osmotic Demyelination Syndrome (ODS) was defined as a neurological complication resulting from rapid correction of sodium, diagnosed based on clinical presentation and confirmed via MRI findings or ICD-10 coding [21]. Readmission was defined as any unplanned hospitalization at NYPQ within 90 days of discharge. Skilled Nursing Facility (SNF) Placement referred to discharge to an extended-care facility, including subacute rehabilitation (SAR) or a skilled nursing facility (SNF).

Definition of Exposure

The primary exposure variable was the timing of nephrology consultation: early consultation (within 48 hours of admission), late consultation (after 48 hours), and no nephrology consultation.

Study outcomes

Primary outcomes included inpatient mortality, defined as death occurring during hospitalization (including ICU and general medical wards); 30-day and 90-day mortality, defined as death occurring within 30 or 90 days post-discharge; length of hospital stay (LOS), defined as the total duration from admission to discharge; and the rate of sodium correction over a 24-hour period, categorized as slow (<6 mEq/L), average (6-10 mEq/L), or rapid (>10 mEq/L). Secondary outcomes included the need for skilled nursing facility (SNF) placement upon discharge, defined as discharge to subacute rehabilitation (SAR), SNF, or other extended-care facilities; readmission rates within 90 days, defined as any unplanned readmission to NYPQ within 90 days; recurrence of hyponatremia upon readmission, defined as serum sodium <130 mmol/L at the time of readmission; and neurological complications within 90 days post-discharge, defined as osmotic demyelination syndrome (ODS), confirmed via imaging or ICD-10 coding.

Statistical analysis

Continuous variables were summarized as medians with interquartile ranges (IQRs), and categorical variables as frequencies and percentages. Patient characteristics were compared across the three groups: early nephrology consultation, late consultation, and no consultation, using the Kruskal-Wallis test for continuous variables and the chi-squared or Fisher’s exact test for categorical variables, as appropriate. Statistical significance was defined as a two-sided p-value < 0.05.

Multivariable logistic regression models were used to assess the independent association of nephrology consultation with binary outcomes, including inpatient mortality, 30-day mortality, 90-day mortality, 30-day readmission, 90-day readmission, and discharge disposition. Covariates included in the models were age, race, baseline serum sodium level, admitting unit, and number of comorbidities.

Multivariable linear regression models were used to analyze continuous outcomes, including length of stay, rate of sodium correction in the first 24 hours, and rate of correction in the subsequent 24 hours, adjusting for the same covariates. Kaplan-Meier survival curves were constructed for 30-day and 90-day mortality and compared using the log-rank test. A Bonferroni correction was applied to account for multiple comparisons among exploratory outcomes. All statistical analyses were conducted using R version 4.5.0 (R Foundation for Statistical Computing, Vienna, Austria).

## Results

Patient characteristics

As shown in Table 1, a total of 423 patients were included in the study. Of these, 173 patients (41%) received a nephrology consultation within 48 hours (early consultation group), 83 patients (20%) received a consultation after 48 hours (late consultation group), and 167 patients (39%) did not receive a nephrology consultation. The median age of the cohort was 76 years (IQR 64-84), and 220 (52%) were female. The most prevalent comorbidities were hypertension 294 (70%), hyperlipidemia 143 (34%), diabetes mellitus 126 (30%), and coronary artery disease 81 (19%). Patients in the early consultation group were older (median age 78 vs. 73 years in the no-consultation group) and had a higher burden of comorbidities, including a greater prevalence of hypertension (46% vs. 35%) and hyperlipidemia (44% vs. 41%). Neurological symptoms such as dizziness, syncope, or lightheadedness were the most common presenting complaints for hyponatremia in 118 (28%), followed by cardiorespiratory symptoms such as shortness of breath, chest pain, or cough in 103 (22%). Sodium levels <120 mEq/L were observed in 53 (53%) of the early consultation group, whereas 145 (45%) of the no-consultation group had sodium levels ≥120 mEq/L. Serum osmolality was measured in 362 (85.6%) of patients, urine osmolality in 332 (78.5%), and urine sodium in 269 (63.6%). The most common etiologies of hyponatremia were syndrome of inappropriate antidiuretic hormone secretion (SIADH) in 142 (34%) of cases and solute-limited intake in 125 (30%). Despite nephrology being consulted in nearly two-thirds of all cases, 39 (44%) of ICU patients with hyponatremia did not receive a nephrology consultation. In terms of treatment, 64 (89%) of hypertonic saline (HTS) used and 11 (100%) of tolvaptan prescriptions were driven by the nephrology team. The most frequent rate of sodium correction was slow (<6 mEq/L/day), observed in 260 (61.4%) of patients during the first 24 hours and 336 (79.4%) during the subsequent 24 hours. In cases where sodium correction exceeded the recommended rate, nephrology was involved in approximately two-thirds of the cases. This was often in response to concerns about overcorrection, with dextrose-containing intravenous (IV) fluids and desmopressin used in 56 (51%) and 19 (61%) of the early consultation group, compared to 33 (30%) and five (16%) in the no-consultation group, respectively.

**Table 1:** Descriptive Characteristics of Patients by Nephrology Consultation Status ^1^Median (interquartile range (IQR)); n (%)

Characteristic	Overall, N = 423^1^	Early Consultation N = 173^1^	Late Consultation N= 83^1^	No Consultation, N = 167^1^
Age	76 (64, 84)	78 (69, 84)	77 (64, 86)	73 (60, 81)
Sex				
Female (F)	220 (52%)	92 (42%)	42 (19%)	86 (39%)
Male (M)	203 (48%)	81 (40%)	41 (20%)	81 (40%)
Ethnicity				
Hispanic	74 (17%)	22 (30%)	19 (26%)	33 (45%)
Not Hispanic	343 (81%)	148 (43%)	63 (18%)	132(38%)
Declined	6 (2%)	3 (50%)	1 (17%)	2 (33%)
Race				
Asian	207 (49%)	94 (45%)	32 (15%)	81 (39%)
Black	16 (3.8%)	8 (50%)	3 (19%)	5 (31%)
Hispanic	4 (0.9%)	1 (25%)	2 (50%)	1 (25%)
White	106 (25%)	40 (38%)	22 (21%)	44 (42%)
Declined	4 (0.9%)	2 (50%)	0 (0%)	2 (50%)
Other	86 (20%)	28 (33%)	24 (28%)	34 (40%)
Comorbidities				
Chronic kidney disease (CKD) (stage 1 or 2)	57 (13%)	29 (51%)	12 (21%)	16 (28%)
Hyperlipidemia (HLD)	143 (34%)	63 (44%)	21 (15%)	59 (41%)
Coronary artery disease (CAD)	81 (19%)	39 (48%)	13 (16%)	29 (36%)
Heart Failure (HF)	44 (10%)	17 (39%)	7 (16%)	20 (45%)
Atrial fibrillation (AF)	52 (12%)	19 (37%)	14 (27%)	19 (37%)
Peripheral arterial disease (PAD)	6 (1.4%)	4 (67%)	0 (0%)	2 (33%)
Cirrhosis	36 (8.5%)	14 (39%)	9 (25%)	13 (36%)
Diabetes mellitus (DM)	126 (30%)	48 (38%)	27 (21%)	51 (40%)
Hypertension (HTN)	294 (70%)	136 (46%)	54 (18%)	104 (35%)
Hypothyroidism	68 (16%)	31 (46%)	15 (22%)	22 (32%)
Adrenal insufficiency	3 (0.7%)	1 (33%)	1 (33%)	1 (33%)
Dementia	33 (7.8%)	16 (48%)	7 (21%)	10 (30%)
Seizure	23 (5.4%)	9 (39%)	3 (13%)	11 (48%)
Stroke	34 (8.0%)	12 (35%)	9 (26%)	13 (38%)
Depression	32 (7.6%)	20 (63%)	3 (9.4%)	9 (28%)
Anxiety	53 (13%)	20 (38%)	13 (25%)	20 (38%)
Schizophrenia/ Bipolar	12 (2.8%)	3 (25%)	3 (25%)	6 (50%)
Cancer	92 (22%)	44 (48%)	12 (13%)	36 (39%)
Other comorbidities	191 (45%)	76 (40%)	39 (20%)	76 (40%)
Number of comorbidities	3.00 (1.00, 4.00)	3.00 (2.00, 4.00)	2.00 (1.00, 4.00)	2.00 (1.00, 3.00)
Presenting complaint (s)				
Abnormal labs	31 (7.3%)	21 (68%)	2 (6.5%)	8 (26%)
Cardiorespiratory	103 (24%)	43 (42%)	18 (17%)	42 (41%)
Fall/Gait	44 (10%)	16 (36%)	10 (23%)	18 (41%)
Mixed	35 (8.3%)	13 (37%)	6 (17%)	16 (46%)
Neurological	118 (28%)	45 (38%)	27 (23%)	46 (39%)
Other	92 (22%)	35 (38%)	20 (22%)	37 (40%)
Initial Na level (mEq/l)				
<120	100 (24%)	53 (53%)	25 (25%)	22 (22%)
>=120	323 (76%)	120 (37%)	58 (18%)	145 (45%)
Other laboratory tests:				
Initial glucose level (mg/dl)	119 (103, 144)	117 (102, 140)	119 (103, 153)	121 (103, 148)
Initial Serum osmolarity (mOsm/kg)	264 (255, 271)	261 (253, 270)	263 (254, 270)	267 (261, 274)
Not checked	61	15	14	32
Initial urine specific gravity	1.014 (1.009, 1.019)	1.014 (1.009, 1.018)	1.013 (1.009, 1.018)	1.014 (1.009, 1.020)
Not checked	47	15	7	25
Urine Na (sodium) (mEq/L)	53 (33, 84)	53 (36, 78)	69 (34, 89)	50 (30, 84)
Not checked	154	55	24	75
Urine K (potassium) (mEq/L)	31 (17, 46)	33 (20, 47)	27 (17, 43)	29 (16, 47)
Not checked	81	11	11	59
Urine osmolarity (mOsm/kg)	368 (260, 534)	384 (270, 546)	364 (241, 551)	349 (260, 488)
Not checked	91	16	13	62
Thyroid Stimulating Hormone (TSH) (uIU/mL)	1.63 (1.03, 2.62)	1.69 (1.03, 2.71)	1.73 (1.21, 2.54)	1.57 (0.88, 2.59)
Not checked	68	5	7	56
Cortisol (mg/dL)	16 (11, 22)	16 (11, 22)	16 (11, 20)	16 (12, 26)
Not checked	138	20	17	101
Uric acid (mg/dL)	3.80 (2.80, 5.20)	3.90 (2.90, 4.90)	3.50 (2.80, 4.50)	3.95 (2.70, 6.33)
Not checked	141	20	18	103
Cause of hyponatremia				
Solute limited	125 (30%)	44 (35%)	28 (22%)	53 (42%)
Thiazide	34 (8.0%)	21 (62%)	6 (18%)	7 (21%)
Selective Serotonin Reuptake Inhibitors (SSRI) and anticonvulsants	21 (5.0%)	9 (43%)	3 (14%)	9 (43%)
Hypervolemia	46 (11%)	24 (52%)	7 (15%)	15 (33%)
Hypovolemic	78 (18%)	26 (33%)	19 (24%)	33 (42%)
Syndrome of inappropriate antidiuretic hormone secretion (SIADH)	142 (34%)	60 (42%)	31 (22%)	51 (36%)
Other	93 (22%)	28 (30%)	17 (18%)	48 (52%)
Admission unit (ICU vs GMF)				
General internal medicine floor (GMF)	334 (79%)	145 (43%)	61 (18%)	128 (38%)
ICU	89 (21%)	28 (31%)	22 (25%)	39 (44%)
Treatment:				
Isotonic IV Fluid (IVF)	264 (62%)	92 (35%)	48 (18%)	124 (47%)
Fluid restriction	423 (100%)	173 (41%)	83 (20%)	167 (39%)
Hypertonic IVF	72 (17%)	44 (61%)	20 (28%)	8 (11%)
Diuretics	65 (15%)	32 (49%)	9 (14%)	24 (37%)
Urea	106 (25%)	67 (63%)	29 (27%)	10 (9.4%)
Salt tablets	50 (12%)	22 (44%)	12 (24%)	16 (32%)
Tolvaptan	11 (2.6%)	8 (73%)	3 (27%)	0 (0%)
Rate of correction in the 1^st^ 24 hrs. (mEq/l)				
Slow (<6)	260 (61.4%)	111 (43%)	51 (20%)	98(37%)
Average (6-10)	129 (30.4%)	50 (39%)	27 (21%)	52 (40%)
Rapid (>10)	34 (8.2%)	12 (35%)	5 (15%)	17 (50%)
Rate of correction in the following 24 hrs. (mEq/l)				
Slow (<6)	336 (79.4%)	145 (43%)	61 (18%)	130(39%)
Average (6-10)	71 (16.8%)	23 (32%)	20 (28%)	28 (39%)
Rapid (>10)	16 (3.8%)	5 (31%)	2 (13%)	9 (56%)
Use of dextrose IVF	109 (100%)	56 (51%)	20 (18%)	33 (30%)
Use of Desmopressin	31 (100%)	19 (61%)	7 (23%)	5 (16%)
Na surge in 1^st^ 24 hrs.	38 (100%)	19 (50%)	6 (16%)	13 (34%)
Na surge in 2^nd^ 24 hrs.	16 (100%)	8 (50%)	3 (19%)	5 (31%)

Outcomes

Regarding the primary outcomes (as shown in Table 2), inpatient mortality was 11 (2.6%) overall, with no statistically significant differences among the consultation groups (p = 0.6). Similarly, mortality within the first month and within three months did not differ significantly between groups. However, the length of hospital stay was significantly longer in patients who received nephrology consultations. The median length of stay was 7 days (IQR 5-10) in the early consultation group and 8 days (IQR 5-12) in the late consultation group, compared to 5 days (IQR 3-8) in the no-consultation group (p < 0.001). The rate of sodium correction in the first 24 hours and in the subsequent 24 hours did not differ significantly across the groups, with p-values of 0.7 and 0.2, respectively. As for secondary outcomes, no significant differences were observed across groups. The need for discharge to a skilled nursing facility was comparable (p = 0.8). Readmission rates within one month and three months were highest in the late consultation group (18% and 33%, respectively), compared to the early consultation group (13% and 23%) and the no-consultation group (11% and 26%), though these differences were not statistically significant (p = 0.3 and p = 0.2, respectively). The presence of hyponatremia on readmission did not significantly differ among groups (p = 0.6). At discharge, the median serum sodium was 134 mEq/L (IQR 132-137), with no significant differences between groups. Notably, no neurological complications such as osmotic demyelination syndrome were reported in any group.

**Table 2:** Outcomes by Consultation Group Outcomes are presented by the consultation group, and p-values are reported for univariate hypothesis tests. A p-value less than 0.05 was considered statistically significant. ^1^n (%); Median (IQR); ^2^Fisher’s exact test; Pearson’s Chi-squared test; Kruskal-Wallis rank sum test

Characteristic	Overall, N = 423^1^	Early Consultation N = 173^1^	Late Consultation N= 83^1^	No consultation N = 167^1^	p-value^2^
Inpatient mortality	11 (2.6%)	4 (2.3%)	1 (1.2%)	6 (3.6%)	0.6
30-days Mortality	21 (5.0%)	10 (5.8%)	5 (6.0%)	6 (3.6%)	0.6
90-days Mortality	17 (4.0%)	6 (3.5%)	5 (6.0%)	6 (3.6%)	0.6
Length of stay (number of days)	6.0 (4.0, 9.0)	7.0 (5.0, 10.0)	8.0 (5.0, 12.0)	5.0 (3.0, 8.0)	<0.001
Rate of correction in the 1^st^ 24 hrs. (mEq/l)					0.7
Average	129 (30%)	50 (29%)	27 (33%)	52 (31%)	
Fast	34 (8.0%)	12 (6.9%)	5 (6.0%)	17 (10%)	
Slow	260 (61%)	111 (64%)	51 (61%)	98 (59%)	
Rate of correction in the subsequent 24 hrs. (mEq/l)					0.2
Average	71 (17%)	23 (13%)	20 (24%)	28 (17%)	
Fast	16 (3.8%)	5 (2.9%)	2 (2.4%)	9 (5.4%)	
Slow	336 (79%)	145 (84%)	61 (73%)	130 (78%)	
Outcome and need for skilled facility on discharge					0.8
Died	10 (2.4%)	4 (2.3%)	1 (1.2%)	5 (3.0%)	
No	243 (57%)	95 (55%)	51 (61%)	97 (58%)	
Yes	170 (40%)	74 (43%)	31 (37%)	65 (39%)	
Na on discharge	134.0 (132.0, 137.0)	134.0 (132.0, 137.0)	133.0 (131.0, 136.0)	134.0 (132.0, 137.0)	0.15
Readmission within one month	57 (13%)	23 (13%)	15 (18%)	19 (11%)	0.3
Readmission within three months	109 (26%)	39 (23%)	27 (33%)	43 (26%)	0.2
Hyponatremia on readmission	99 (50%)	37 (37%)	25 (26%)	37 (37%)	0.6

Causes of death

As shown in Table 3, among the 423 patients, there were 39 deaths during the follow-up period. Cancer was the most frequently reported cause in 15 patients, representing 38% of all deaths, and remained the leading cause across all consultation groups. Infection and cardiorespiratory causes followed, accounting for nine (23%) and seven (18%) deaths, respectively. Notably, the distribution of causes varied modestly by consultation status. Cancer-related mortality was consistent across groups, while cardiorespiratory deaths were more common in the early consultation group. Infection-related deaths were relatively evenly distributed but slightly more frequent among patients who received early nephrology input. Other causes of death were more prevalent in the no-consultation group.

**Table 3:** Causes of Death by Consultation Group ^1^n (%)

Cause of death	Overall N = 39^1^	Early Consultation N = 17^1^	Late Consultation N = 8^1^	No Consultation N = 14^1^
Cancer	15 (38%)	5 (33%)	4 (27%)	6 (40%)
Cardiorespiratory	7 (18%)	5 (71%)	0 (0%)	2 (29%)
Infection	9 (23%)	5 (56%)	2 (22%)	2 (22%)
Other	8 (21%)	2 (25%)	2 (25%)	4 (50%)

Multivariable analysis of nephrology consultation and clinical outcomes

As shown in Table 4, multivariable logistic regression analysis showed that nephrology consultation was not significantly associated with inpatient mortality, short-term mortality (30- or 90-day), discharge disposition, or hospital readmission. After adjusting for age, race, initial sodium level, admitting unit (GMF vs ICU), and comorbidity burden, none of the evaluated outcomes demonstrated a statistically significant difference between patients who received a nephrology consultation and those who did not.

**Table 4:** Multivariable Logistic Regression Models Assessing the Association Between Nephrology Consultation and Clinical Outcomes, Adjusted for Age, Race, Initial Sodium, Admitting Unit (GMF vs ICU), and Comorbidity Burden ^1^A p-value less than 0.05 was considered statistically significant. GMF: general internal medicine floor; SNF: Skilled Nursing Facility

Term	Outcome	Odds ratio (95% CI)	P-value^1^
Consultation vs no consultation	Inpatient mortality	0.548 (0.117, 2.392)	0.422
Consultation vs no consultation	30-day mortality	1.598 (0.664, 4.168)	0.311
Consultation vs no consultation	90-day mortality	1.383 (0.692, 2.876)	0.369
Consultation vs no consultation	Need for SNF	0.992 (0.644, 1.529)	0.971
Consultation vs no consultation	Readmission in 1 month	1.269 (0.689, 2.396)	0.452
Consultation vs no consultation	Readmission in 3 months	1.047 (0.653, 1.69)	0.850

Multivariable analysis of length of stay and sodium correction trends by consultation status

As shown in Table 5, multivariable linear regression showed that nephrology consultation was significantly associated with a longer hospital stay, with an adjusted mean increase of 2.74 days (95% CI: 1.25-4.23, p<0.001). Although consultation was linked to a slower sodium correction rate in both the first and second 24-hour periods, these differences were not statistically significant after applying Bonferroni correction for multiple comparisons. The negative estimates indicate a trend toward slower correction in the consultation group when controlling for age, race, initial sodium, admitting unit, and comorbidity burden. Outcomes analyzed included length of stay, rate of sodium correction in the first 24 hours, and the subsequent 24 hours. Bonferroni correction was used to reduce the risk of Type I error due to repeated hypothesis testing.

**Table 5:** Multivariable Linear Regression Models for Length of Stay and Rate of Sodium Correction, Adjusted for Age, Race, Initial Sodium, Admitting Unit, and Comorbidities ^1^A p-value less than 0.05 was considered statistically significant.

Term	Outcome	Regression estimate (95% CI)	P-value	Adjusted p-value ^1^
Consultation vs no consultation	Length of stay (number of days)	2.74 (1.248, 4.232)	<0.001	<0.001
Consultation vs no consultation	Rate of correction in the 1^st^ 24 hrs.	-0.886 (-1.703, -0.069)	0.034	0.102
Consultation vs no consultation	Rate of correction in the subsequent 24 hrs.	-0.784 (-1.622, 0.053)	0.067	0.201

## Discussion

Consistent with prior studies, hyponatremia in our cohort was predominantly observed among elderly patients with a substantial burden of comorbidities. Lindner et al. demonstrated a significant decline in serum sodium levels with increasing age [22], while Kayar et al. reported a higher risk of moderate to severe hyponatremia with an increasing number of comorbidities [23]. SIADH and solute-limited intake were the most common etiologies identified in our population, aligning with findings by Adrogué et al., who highlighted thiazide use, postoperative states, and other SIADH-related causes as leading contributors to severe hyponatremia [24]. In contrast, Ande et al. identified postoperative conditions and gastroenteritis as predominant causes, with most participants lacking comorbidities [25], underscoring variability based on population characteristics.

Nephrology involvement was relatively common in our study, with nearly 60% of patients receiving consultation during hospitalization. However, 39% did not receive nephrology input, including 44% of ICU admissions. This suggests variability in consultation practices and potential under-recognition of nephrology’s role in managing complex electrolyte disorders.

Notably, nephrology consultation was not associated with improved primary outcomes. Inpatient mortality was low overall (2.6%) and showed no significant difference between consultation groups. Similarly, 30- and 90-day mortality rates were unaffected by the presence or timing of consultation. These findings persisted after multivariable logistic regression, adjusting for confounders such as age, race, initial sodium level, comorbidity burden, and admission unit. These results contrast with an earlier study suggesting a mortality benefit from nephrology involvement, particularly in critically ill patients or those with severe hyponatremia [18].

One possible explanation is the relatively mild-to-moderate hyponatremia severity in our cohort, with a large proportion having sodium levels ≥120 mEq/L. The association between hyponatremia and mortality has been well documented. Holland-Bill et al. found a stepwise increase in 30-day mortality with decreasing sodium levels, from 3.6% at normal levels to 9.6% at levels <120 mmol/L [26]. Similarly, Waikar et al. demonstrated increased in-hospital and long-term mortality with sodium levels of 130-134 mEq/L (24% increase) and 125-129 mEq/L (33% increase) [27]. A meta-analysis by Corona et al. confirmed this relationship, reporting a 2.6-fold increase in mortality risk associated with hyponatremia across various conditions, including myocardial infarction and heart failure [28].

Mannheimer et al. (2025) reinforced these findings, showing increased 30-day mortality with hyponatremia severity (HRs 1.35 to 3.38), though longer-term mortality was mainly attributable to malignancy and gastrointestinal disease, suggesting hyponatremia may be a marker of underlying illness severity rather than a direct cause [29]. Chawla et al. found that while mortality increased as sodium dropped to 120 mEq/L, it paradoxically decreased below that level. Most deaths occurred in patients with severe comorbidities, and only a small fraction were directly attributable to hyponatremia itself, reinforcing the idea that comorbidities play a dominant role in outcomes [30].

Interestingly, despite the lack of mortality benefit, nephrology consultation was significantly associated with prolonged hospital stay. Our multivariable analysis showed an adjusted increase of 2.74 days in the consultation group. This may reflect more extensive workups, closer monitoring, or use of interventions such as hypertonic saline, desmopressin, and IV fluids - more frequently used among those seen by nephrology. Delays from late consultation could also contribute. Alternatively, consultation may have been more common in clinically complex cases, introducing confounding by indication. These findings contrast with Herrera et al., who found earlier nephrology referral reduced hospital stay [18].

While sodium correction was slower in the consultation group during the first and second 24-hour periods, these differences were not statistically significant after Bonferroni correction. This may suggest that nephrology teams are often consulted when sodium levels are refractory to initial treatment. These findings align with consensus guidelines recommending gradual correction to avoid osmotic demyelination syndrome (ODS) [20]. Encouragingly, no cases of ODS or other neurological complications were reported across consultation groups. However, recent studies have raised concerns that overly slow correction may be associated with increased mortality [2, 5, 9, 10].

Secondary outcomes, including hospital readmission and discharge disposition, did not differ significantly by consultation status. One- and three-month readmission rates were slightly higher in the late consultation group but lacked statistical significance. Similarly, skilled nursing facility (SNF) placement was not influenced by consultation. These findings imply that while nephrology may impact inpatient management, it may not significantly alter post-discharge outcomes. However, readmissions may have been driven by non-electrolyte-related issues, particularly malignancy, which was the most frequent cause of death in our cohort. Prior literature supports the association between hyponatremia and increased risks of falls, fractures, and adverse outcomes, especially in patients with functional or cognitive impairment [1,2].

Hyponatremia is a known risk factor for readmission, particularly when the underlying cause is unresolved or when sodium correction is suboptimal. In our study, likely multifactorial etiologies - including volume depletion, heart failure, and SIADH - created a clinical scenario in which maintaining sodium balance post-discharge was challenging. Studies have shown that inadequate correction and lack of follow-up are major contributors to early readmissions [15]. This underscores the importance of tailored discharge planning and appropriate outpatient follow-up. Kutz et al. found that hyponatremia significantly increased the risk of short-term adverse outcomes, particularly readmission [31]. Deitelzweig et al. also demonstrated that among heart failure patients, hyponatremia was linked to higher mortality and increased 30-, 90-, and 180-day readmission rates [32].

Finally, our cause-of-death analysis revealed that cancer was the leading cause, followed by infections and cardiopulmonary complications. This distribution was consistent across consultation groups, suggesting that the primary drivers of mortality were often unrelated to electrolyte disturbances. This further supports the idea that nephrology consultation may have limited influence on mortality in populations where underlying conditions - not hyponatremia - are the principal determinants of outcome [2].

This study is limited by its retrospective design, which introduces potential for selection bias and unmeasured confounding, especially regarding the decision to request nephrology input. It was conducted at a single tertiary-care center, which may limit the generalizability of findings. The severity of illness was estimated based on clinical variables rather than formal scoring systems. Moreover, the timing and content of nephrology consultations were not standardized or consistently recorded, precluding detailed assessment of intervention quality. Lastly, long-term outcomes beyond 90 days and post-discharge follow-up adherence were not evaluated.

## Conclusions

This retrospective study demonstrated that nephrology consultation in hospitalized patients with hyponatremia was not significantly associated with improved inpatient or post-discharge outcomes, including mortality, readmission, or discharge disposition. Nephrology was more frequently consulted in patients with more complex clinical presentations, such as refractory or severe hyponatremia, need for hypertonic therapy, or concern for overcorrection. These cases also had longer hospital stays and a trend toward slower sodium correction, likely reflecting the higher illness acuity and need for close monitoring. Despite this, no neurological complications such as osmotic demyelination syndrome were observed in any group, suggesting adherence to safe correction protocols by both primary teams and nephrologists. The absence of a mortality difference implies that short-term outcomes are more closely linked to underlying disease burden than the presence or timing of nephrology involvement. These findings underscore the selective but valuable role of nephrology in guiding individualized therapy, particularly in medically complex patients.
